# Lay Service in Ambulance Work

**Published:** 1920-11-20

**Authors:** 


### Lay Service in Ambulance Work.

INSPECTOR-GENERAL H. C. WOODS, R.N., has retired from the post of Chairman to the Gosport Centre of the St. John Ambulance Association after thirty-eight years' work. Born in 1841, the retiring Chairman, who is honorary physician to the King, has been Inspector-General of Hospitals and the Fleet for many years. The Treasurer, Mr. W. M. Clay, is also retiring, with twenty-three years' work in this office to his credit. The new Chairman is the Hon. Major-General W. Huskisson, C.M.G., who has been, among other things, Professor of Fortifications at the R.M. College, Kingston, Canada. Miss Paine succeeds Mr. W. M. Clay in the treasurership.

